# High Risk, Low Key: The New Face of Drug Use

**DOI:** 10.1177/29768357251389682

**Published:** 2025-10-26

**Authors:** James R. Burmeister, John K. Jung

**Affiliations:** 1 Department of Foundational Medical Studies, Oakland University William Beaumont School of Medicine, Rochester, MI, USA; 2 John Sealy School of Medicine, University of Texas Medical Branch, Galveston, USA

**Keywords:** modern drug use, cannabis rebranding, self-medication, nitrous oxide abuse, fentanyl crisis, wellness marketing, adolescent substance use, digital drug markets

## Abstract

In today’s rapidly evolving substance use landscape, the traditional image of drug consumption is being replaced by a subtler, more socially accepted aesthetic. High Risk, Low Key: The New Face of Drug Use explores how modern intoxicants, from THC-infused beverages to microdosed psilocybin and nitrous oxide canisters, are increasingly marketed as wellness products rather than recreational drugs. This shift, driven by cultural, technological, and mental health trends, has led to the normalization and concealment of daily drug use, especially among youth. Cannabis, once emblematic of rebellion, is now branded as organic and therapeutic, despite rising potency and associated risks. Meanwhile, legal gray-area substances like kratom and nitrous oxide offer easily accessible highs with potentially serious health consequences. The rise of self-medication, fueled by online platforms and a mental health crisis, further blurs the line between therapy and abuse. Digital platforms now serve as decentralized drug markets, contributing to the fentanyl crisis through counterfeit pills. This article calls for a new framework to address these trends, one that includes updated education, regulation, and clinical tools responsive to a generation navigating a silent, rebranded drug crisis.

## Introduction

We’re living in a new era of substance use. The cultural image of “drugs” has changed, no longer associated only with dark alleys, slurred speech, and telltale signs of addiction. Today’s intoxicants come in minimalist cans, fruit-flavored gummies, and vape pens that fit neatly in a hoodie pocket. They’re branded as clean, wellness-adjacent, and even aspirational. From tetrahydrocannabinol (THC)-infused sparkling water to microdosed mushroom chocolates and gas station whip-it canisters, drug use is being rebranded, not eradicated.

This isn’t just a shift in how substances are packaged, it’s a shift in how they’re perceived, accessed, and justified.

## The Wellness Rebrand of Cannabis

Cannabis has completed a full-blown rebrand. No longer the mascot of rebellion, it’s now a lifestyle product. THC is being infused into sodas, seltzers, and granola bars. It’s discreet, attractive, and marketed as “organic,” “natural,” and “non-toxic.”^1^ Based on this marketing, cannabis could be perceived as closer to a vitamin supplement than a psychoactive drug.

Music, fashion, and youth culture contribute to shifting perceptions of cannabis. References to cannabis are now common in popular music and media, presenting its use as routine or aspirational, especially among youth.

But behind the sleek packaging and lyrical hooks is a concerning reality: cannabis is the most commonly abused illicit substance among adolescents in the US.^2^ And today’s cannabis is not what it was in decades past, potency levels have skyrocketed, increasing the risk of dependency and mental health consequences.^3^

While cannabis is widely viewed as a safer alternative to alcohol or hard drugs, chronic use, especially in teens and young adults, is associated with Cannabis Use Disorder, cognitive impairment, and increased risk for Cannabis-Induced Psychosis, particularly in genetically or neurologically vulnerable individuals.^4,5^

Despite this, research into cannabis remains heavily limited. Because it’s still classified as a Schedule I drug at the federal level in the US, researchers face significant barriers to funding, approval, and access to standardized THC for clinical trials.^6^

## Nitrous Oxide and the Illusion of Legality

Once confined to dental offices and hospital settings, nitrous oxide, or “laughing gas”, has quietly entered mainstream youth culture. Nitrous oxide has been used in dentistry for well over a century, valued for its calming and pain-relieving properties alongside traditional reassurance techniques. Along with suggestive hypnosis and reassurance, nitrous oxide is often sufficient to alleviate anxiety.^7^

Sold as “whip-it” canisters in gas stations and online, nitrous is legally marketed as a kitchen tool for whipped cream. But for many, it’s a cheap, quick, accessible high. Inhaling nitrous repeatedly can deplete vitamin B12, causing neurological damage, memory loss, or even paralysis.^8^ In the UK, clinician enquiries to the National Poisons Information Service (NPIS) concerning nitrous oxide rose from 15 in 2018 to 85 in 2022, a nearly 500 % increase, though no national data confirms a similar rise in actual hospital admissions.^9^ Inhalant use, including nitrous oxide, remains most common among adolescents, with around 4% of 8th graders reporting past-year use, according to the latest NIDA-funded Monitoring the Future survey.^10^

Nitrous oxide is legal in the US for culinary use, but the Food and Drug Administration (FDA) prohibits its sale for human consumption under misbranding rules.^11^ In the US, there is no federal law establishing a minimum age for the purchase of nitrous oxide, leaving regulation primarily to the states. Many states have implemented restrictions on the sale of nitrous oxide, commonly setting the minimum purchasing age at 18. However, regulation remains highly fragmented in the absence of a federal minimum age requirement.^12^

## Mental Health, Diagnosis, and the Age of Self-Medication

Another force driving new drug trends is the mental health crisis, and the shifting attitudes around it. Rates of anxiety and depression are rising among youth. According to the Centers for Disease Control and Prevention (CDC), 29% of high school students reported experiencing poor mental health during the past year.^13^ While increased diagnosis may partly reflect better awareness and de-stigmatization, it’s also a sign of serious systemic stress: social media pressures, financial insecurity, academic burnout, and climate anxiety all play a role.^14^

In response, many are turning to substances that are marketed as tools for healing or self-care, rather than as dangerous or recreational drugs. THC for anxiety. Kratom for pain. Psilocybin microdoses for productivity and mood stabilization. This is Do It Yourself (DIY) psychiatry, driven by podcasts, social media testimonials, and peer-to-peer anecdotes.^15^

But without clinical oversight, self-medicating can mask deeper issues or spiral into dependency. The line between “wellness hack” and “substance use disorder” is increasingly blurred, especially when the products don’t come with warning labels, dosage standards, or meaningful regulation.

## Kratom, Mushrooms, and the New Herbal Gray Zone

Distrust of traditional prescription medications, combined with barriers to access such as cost, stigma, or lack of providers, has pushed some individuals toward alternative substances. When prescription solutions feel out of reach, cannabis, kratom, or psilocybin may appear to be more accessible “natural” options.^16,17^ Kratom, for example, according to the National Institutes of Health (NIH), is a plant-based stimulant with opioid-like effects.^18^ Marketed as a natural supplement, it’s sold in smoke shops, gas stations, and online, sometimes with ambiguous labeling to avoid regulation.^19^

Kratom’s growing popularity in the US can be attributed to its legal ambiguity, ease of access, and perceived natural origins. In the wake of the opioid crisis and increasing distrust in pharmaceutical companies, many users view kratom as a “safer” alternative for managing pain, anxiety, or fatigue. Its ability to produce both stimulating and sedative effects, depending on dosage, makes it appealing for a range of uses. Like cannabis and nitrous oxide, kratom is easily accessible, especially to teens seeking a “legal high.”^19^

## Motivations Matter: Self-Medication Versus Recreation

Not all substance use is driven by the same motivations. While cannabis, nitrous oxide, and kratom are often framed as recreational or social drugs, other substances such as psilocybin microdoses, adaptogens, and nootropics are increasingly adopted for self-medication or performance enhancement. Distinguishing between recreational motivations and self-treatment is critical for clinicians, since each pathway carries different risks, perceptions, and intervention opportunities.

The mushroom microdosing movement, for instance, is an increasingly popular practice where users take sub-hallucinogenic doses of psilocybin to boost creativity, reduce anxiety, or improve focus.^20^ While there’s promising research in clinical settings, the mainstream adoption of psilocybin often skips the science and goes straight to online shopping carts.

Add to that lion’s mane mushroom, nootropics, adaptogens, and herbal extracts, all marketed as cognitive enhancers, and you get a market flooded with brain-altering substances that have little regulation and even less long-term research behind them. While marketed as safe and natural, these substances may cause adverse effects or interact with medications, especially when used without guidance.^21,22^

There is emerging clinical research suggesting that psilocybin-assisted therapy may help treat addiction and other mental health disorders, but these outcomes are observed under controlled, therapeutic conditions.^23^ Using psilocybin or kratom informally to mitigate harms from illicit drugs remains risky and unproven. Without proper dosing, guidance, or medical oversight, such use may compound harms rather than reduce them.

## Digital Dealers and the Fentanyl Pipeline

One of the most chilling shifts is how people are buying drugs. Social media apps like Snapchat and Telegram have become modern-day drug markets.^24,25^ Deals go down through disappearing messages, emojis, and nicknames. The anonymity and speed of these platforms make it nearly impossible for law enforcement, or parents, to track what’s happening.

Payments often happen via cryptocurrency or peer-to-peer apps like CashApp or Venmo, usually coded in ways to avoid detection.^26^ Users are receiving counterfeit pills laced with fentanyl, which can be lethal even in micrograms. According to the National Institute on Drug Abuse (NIDA), in 2023, the U.S. saw over 72,000 deaths attributed to synthetic opioids other than methadone, primarily illicitly manufactured fentanyl.^27^

Young people are unknowingly consuming substances that look like common meds, Adderall, Xanax, Percocet, but are deadly. With no oversight and no warning, the digital drug economy is fueling a silent epidemic.

## The Rise of Concealable, Daily-Use Substances

Unlike traditional drugs that leave obvious marks, smell, needles, bottles, modern substances are subtle, concealable, and often odorless. THC vapes don’t leave a trail. Microdoses don’t cause visible intoxication. Kratom looks like tea. A whip-it leaves no trace. This discretion makes them harder to detect, by parents, employers, educators, or even peers.

This new era of “silent highs” is particularly concerning in youth and school environments. Vaping devices for nicotine and cannabis are increasingly favored among youth because they are easy to conceal in school settings.^28,29^ In response, many schools have adopted high-tech interventions, including vape sensors and trained detection dogs.^30^

## A New Framework for a New Generation of Drugs

We don’t need a new war on drugs. We need a new framework: modernized history-taking and drug screening tools that include herbal and synthetic substances; education that is honest, evidence-based, and nuanced; early and accessible psychiatric care that addresses root causes; regulation that catches up with wellness-marketed gray-zone substances; and policies that recognize the role of tech in facilitating drug use and distribution.

Public awareness must evolve to match the aesthetics and platforms of modern drug use. Health communication strategies should prioritize digital fluency, leveraging short-form video platforms like TikTok, YouTube Shorts, and Instagram to disseminate harm-reduction messaging. Collaborations with youth influencers, mental health advocates, and creators can help normalize conversations about risk while dismantling misinformation and stigma.

Substance use isn’t disappearing. It’s evolving. It’s quieter, cleaner, and often more deceptive. If we don’t adjust our awareness, our education, and our policies to meet the moment, we risk letting a new generation fall into a different kind of drug crisis, one hidden behind clean branding and a false sense of safety.

## Conclusion

The substances may have changed, but the stakes remain high. As drug use blends into daily life through wellness language, sleek branding, and digital anonymity, we must reframe our response. Prevention and intervention need to be data-driven, clinically informed, and youth-centered. Failing to recognize this rebrand risks letting an invisible epidemic grow louder beneath the silence.
